# Supplementary figures and images for: Telomerase and alternative lengthening of telomeres coexist in the regenerating zebrafish caudal fins
Source: EMBO Rep. 2025 Oct 21;26(23):5776–98. doi: 10.1038/s44319-025-00602-6 (PMC12678820; doi:10.1038/s44319-025-00602-6)

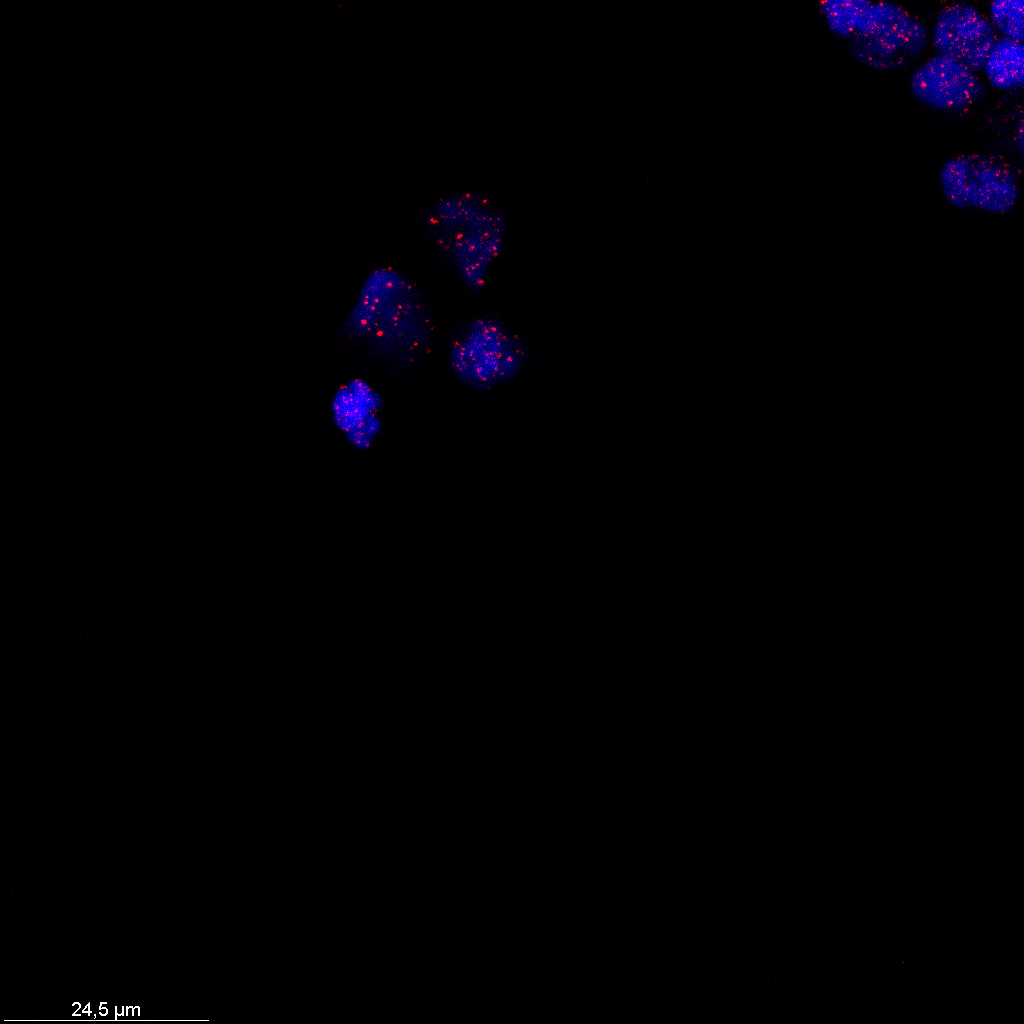

Supplement: Supplementary file 4 — Source data Fig. 3 [file 44319_2025_602_MOESM4_ESM.zip › Figure3/3B/3B down right.tif]

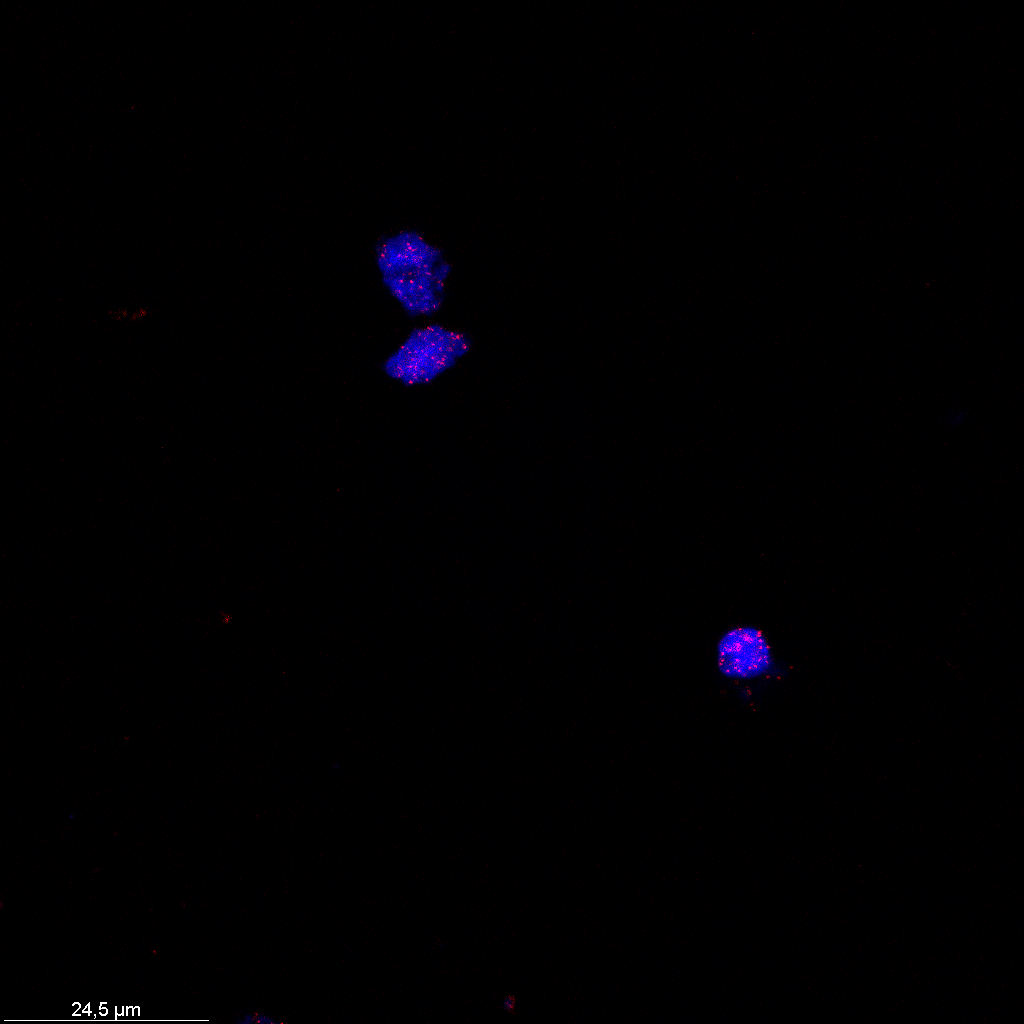

Supplement: Supplementary file 4 — Source data Fig. 3 [file 44319_2025_602_MOESM4_ESM.zip › Figure3/3B/3B up right.tif]

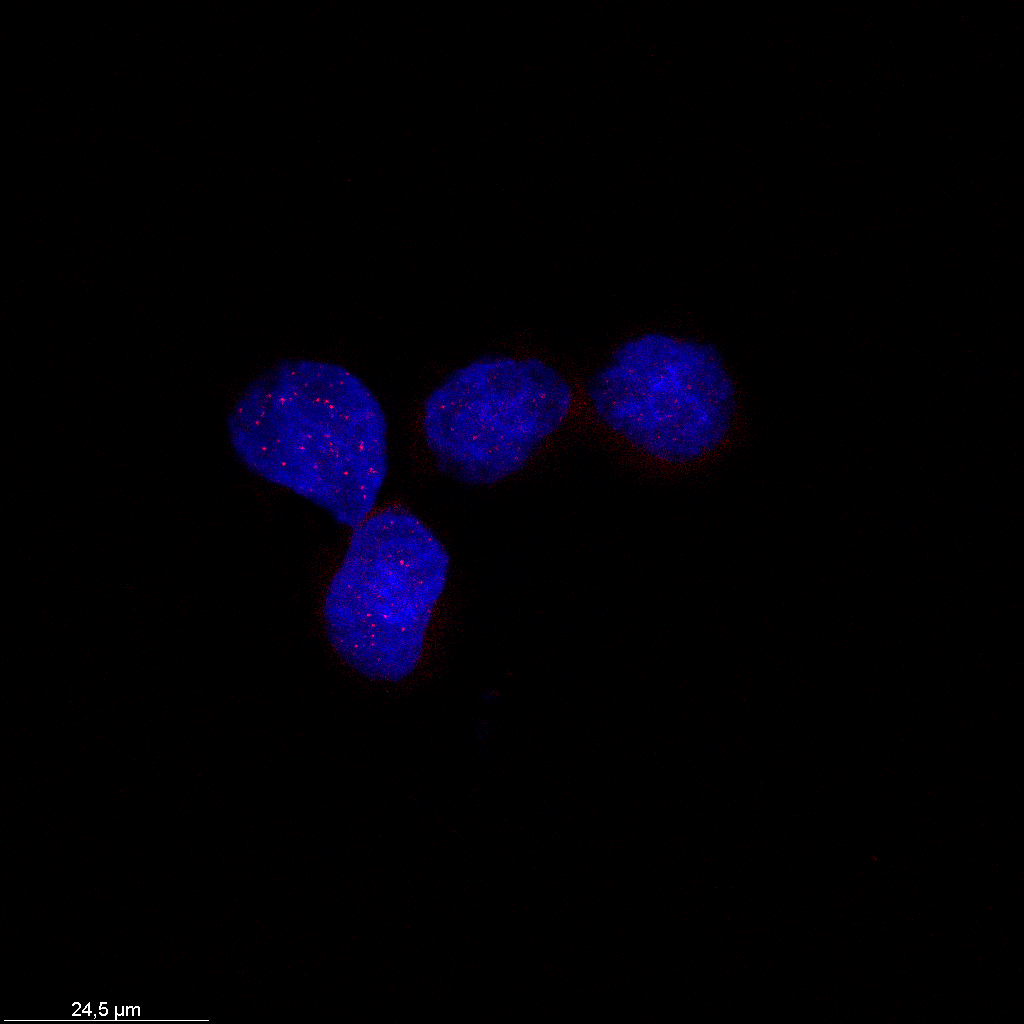

Supplement: Supplementary file 4 — Source data Fig. 3 [file 44319_2025_602_MOESM4_ESM.zip › Figure3/3B/3B down left.tif]

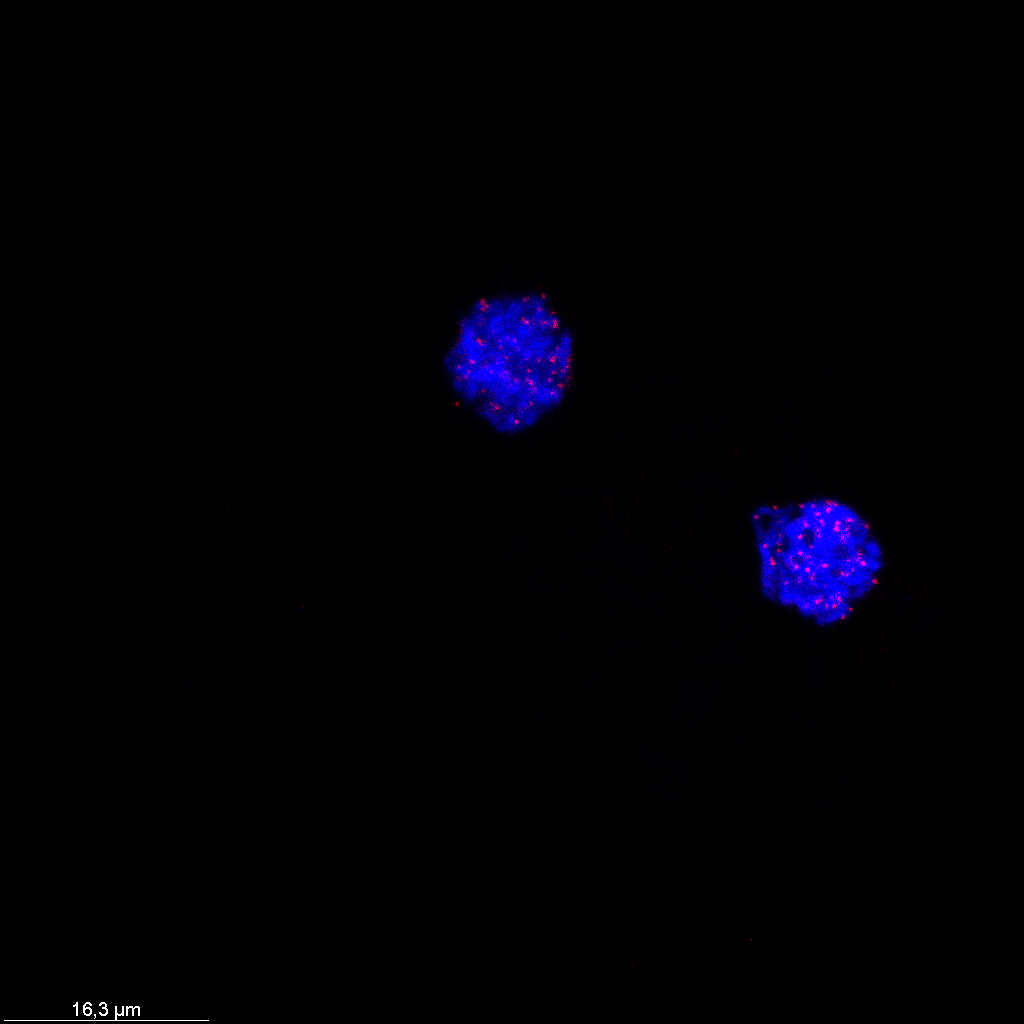

Supplement: Supplementary file 4 — Source data Fig. 3 [file 44319_2025_602_MOESM4_ESM.zip › Figure3/3B/3B up left.tif]

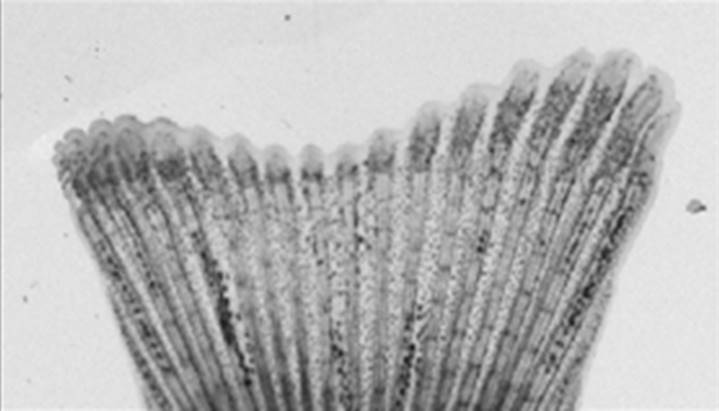

Supplement: Supplementary file 6 — Source data Fig. 5 [file 44319_2025_602_MOESM6_ESM.zip › Figure5/panel B/panel B Mo-atr ter--.jpg]

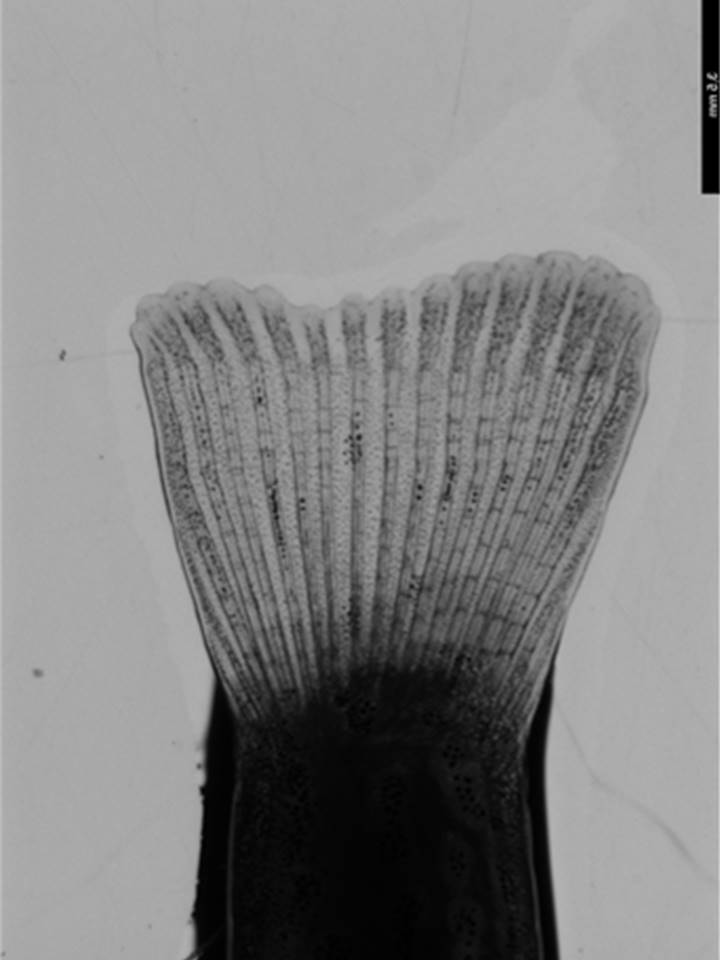

Supplement: Supplementary file 6 — Source data Fig. 5 [file 44319_2025_602_MOESM6_ESM.zip › Figure5/panel B/panel B Mo-atr tert++.jpg]

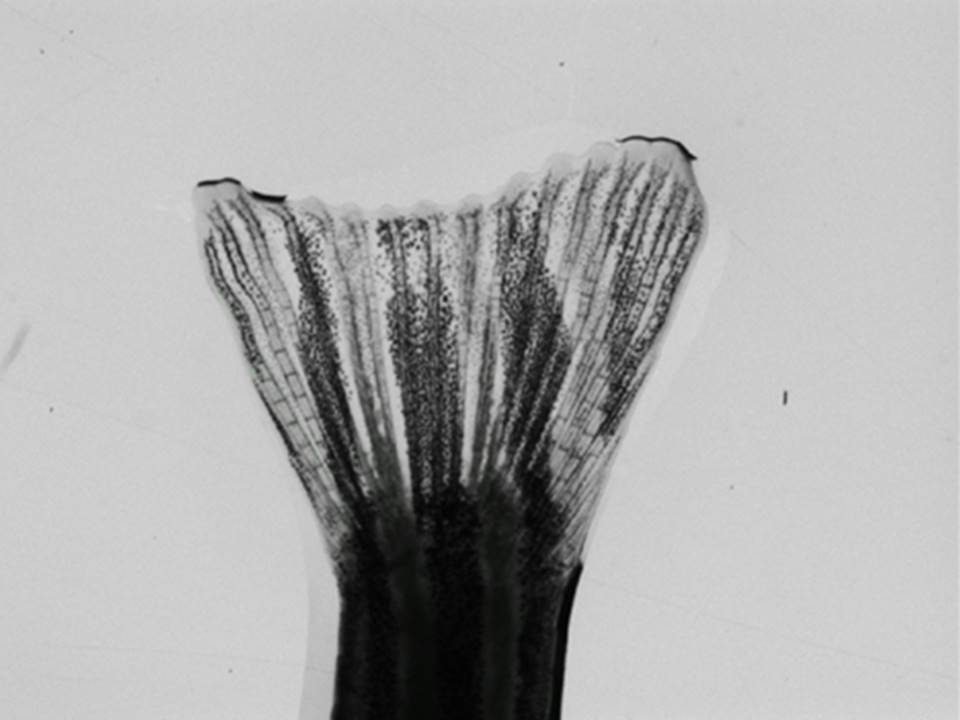

Supplement: Supplementary file 6 — Source data Fig. 5 [file 44319_2025_602_MOESM6_ESM.zip › Figure5/panel B/panel B Mo-nbs1 tert--.jpg]

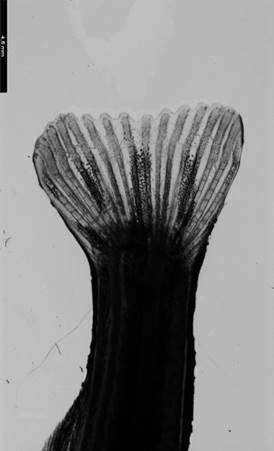

Supplement: Supplementary file 6 — Source data Fig. 5 [file 44319_2025_602_MOESM6_ESM.zip › Figure5/panel B/panel B Mostd tert++.jpg]

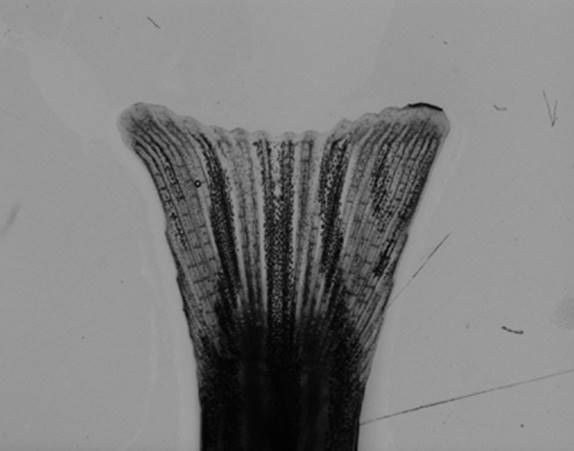

Supplement: Supplementary file 6 — Source data Fig. 5 [file 44319_2025_602_MOESM6_ESM.zip › Figure5/panel B/panel B Mostd tert--.jpg]

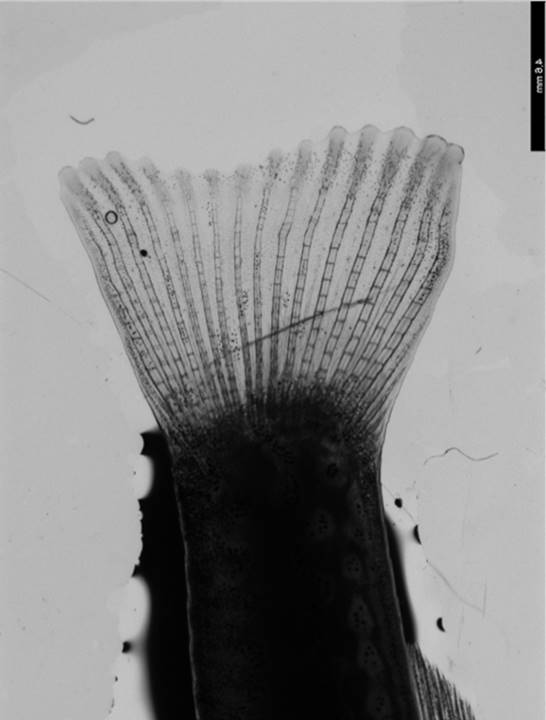

Supplement: Supplementary file 6 — Source data Fig. 5 [file 44319_2025_602_MOESM6_ESM.zip › Figure5/panel B/panel B Mo-nbs1 tert++.jpg]

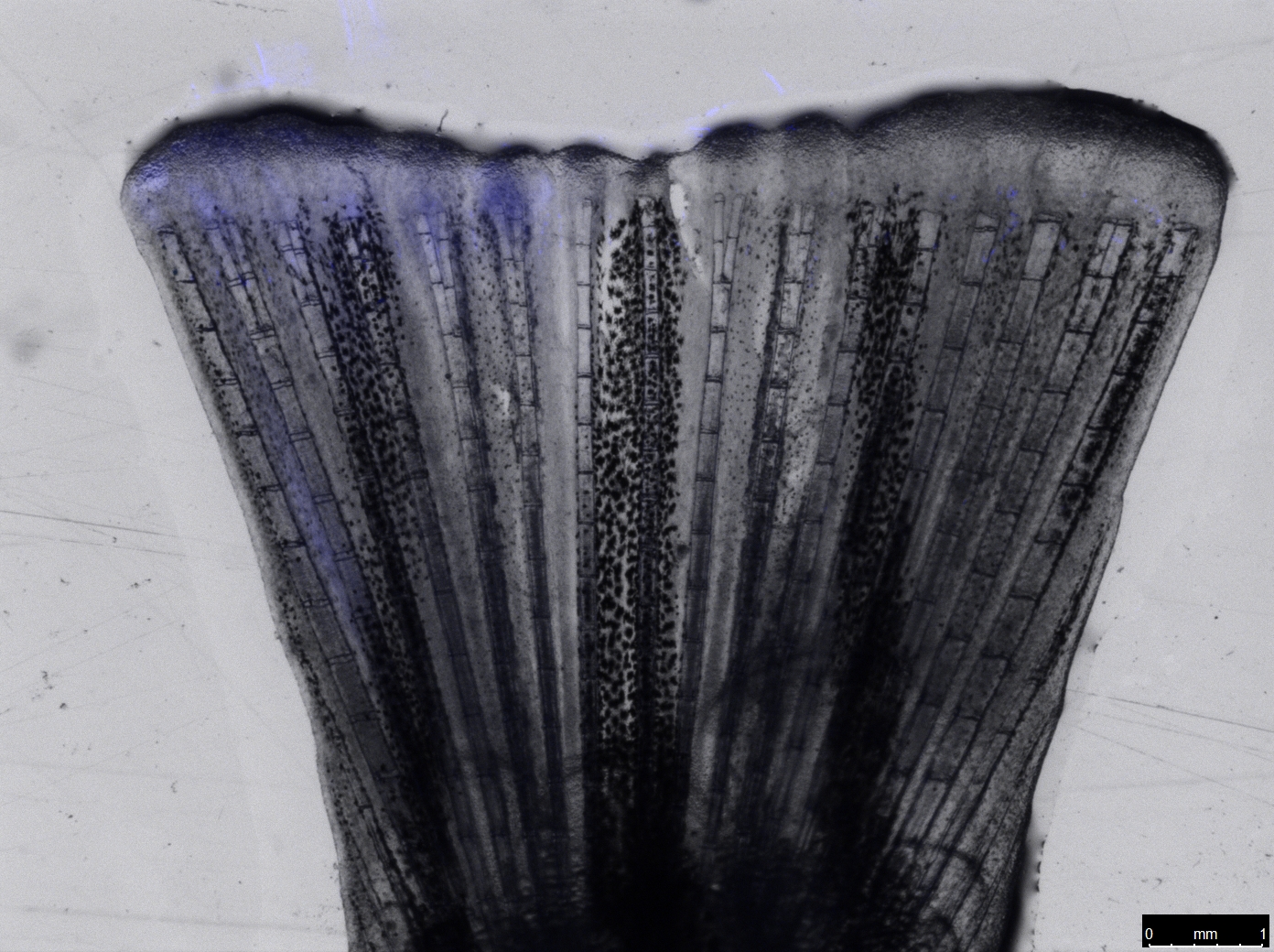

Supplement: Supplementary file 6 — Source data Fig. 5 [file 44319_2025_602_MOESM6_ESM.zip › Figure5/panel D/tert0dpi.jpg]

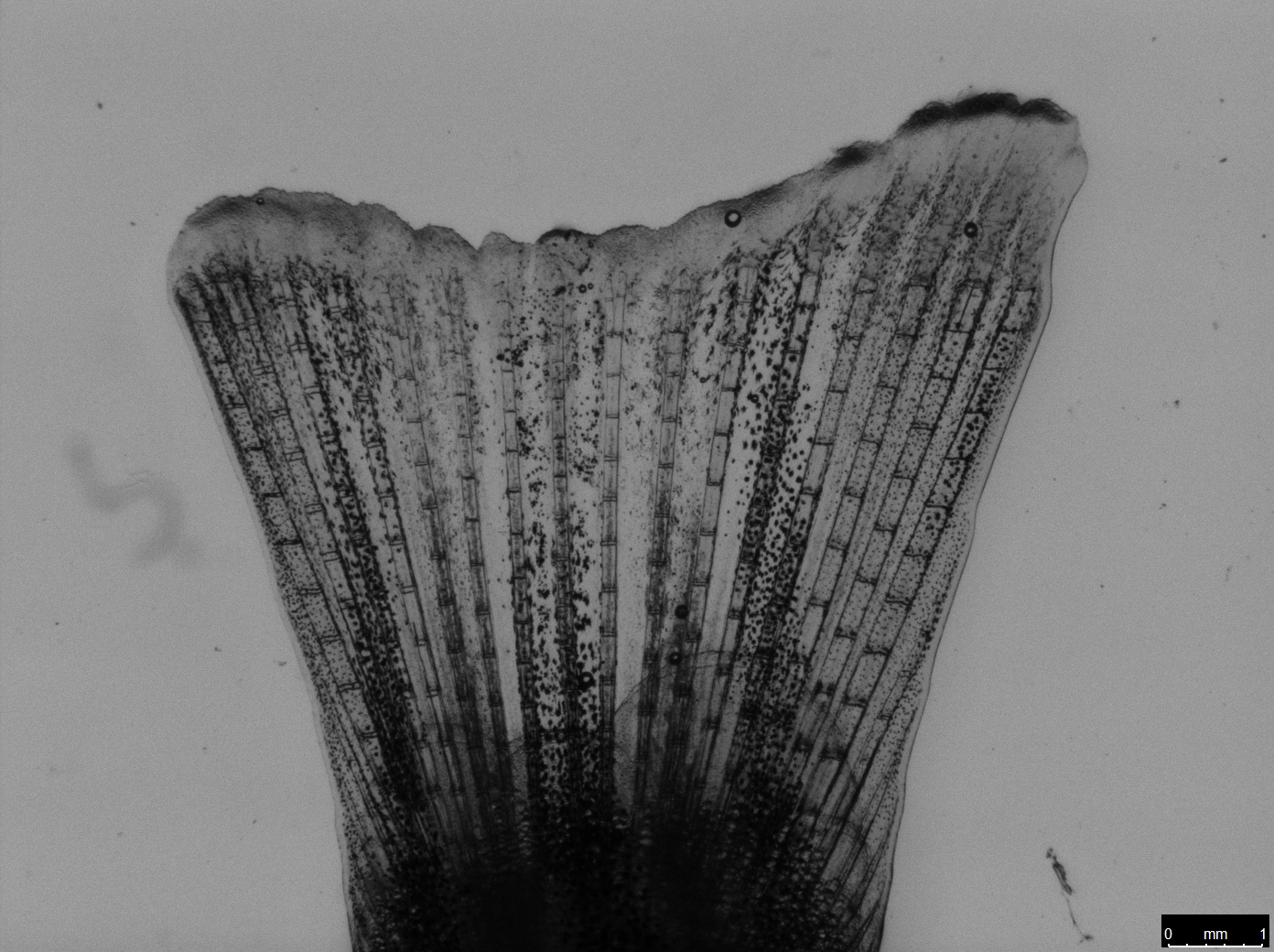

Supplement: Supplementary file 6 — Source data Fig. 5 [file 44319_2025_602_MOESM6_ESM.zip › Figure5/panel D/Mo tert 48hpi.jpg]

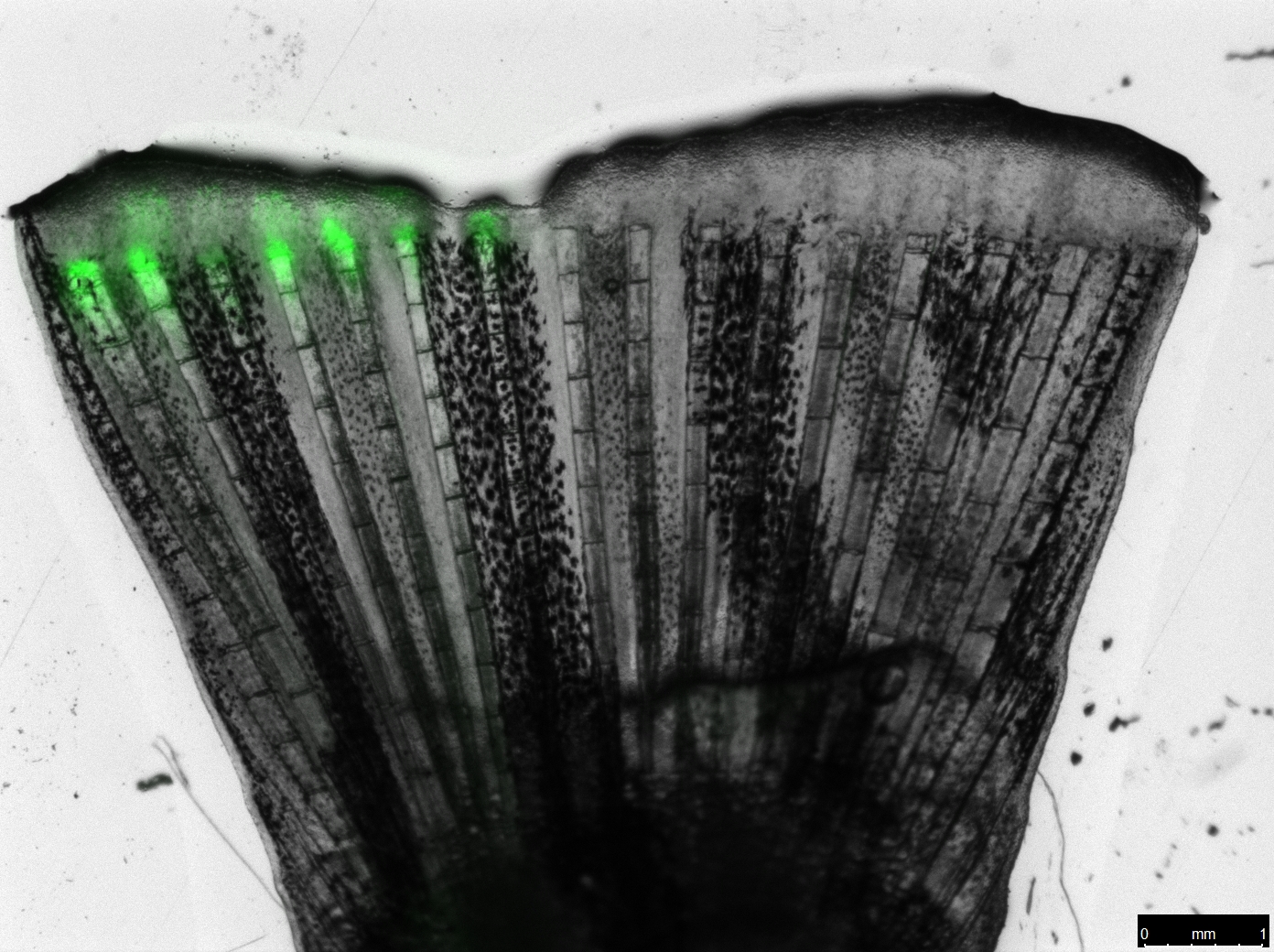

Supplement: Supplementary file 6 — Source data Fig. 5 [file 44319_2025_602_MOESM6_ESM.zip › Figure5/panel D/Mo atr 0dpi.jpg]

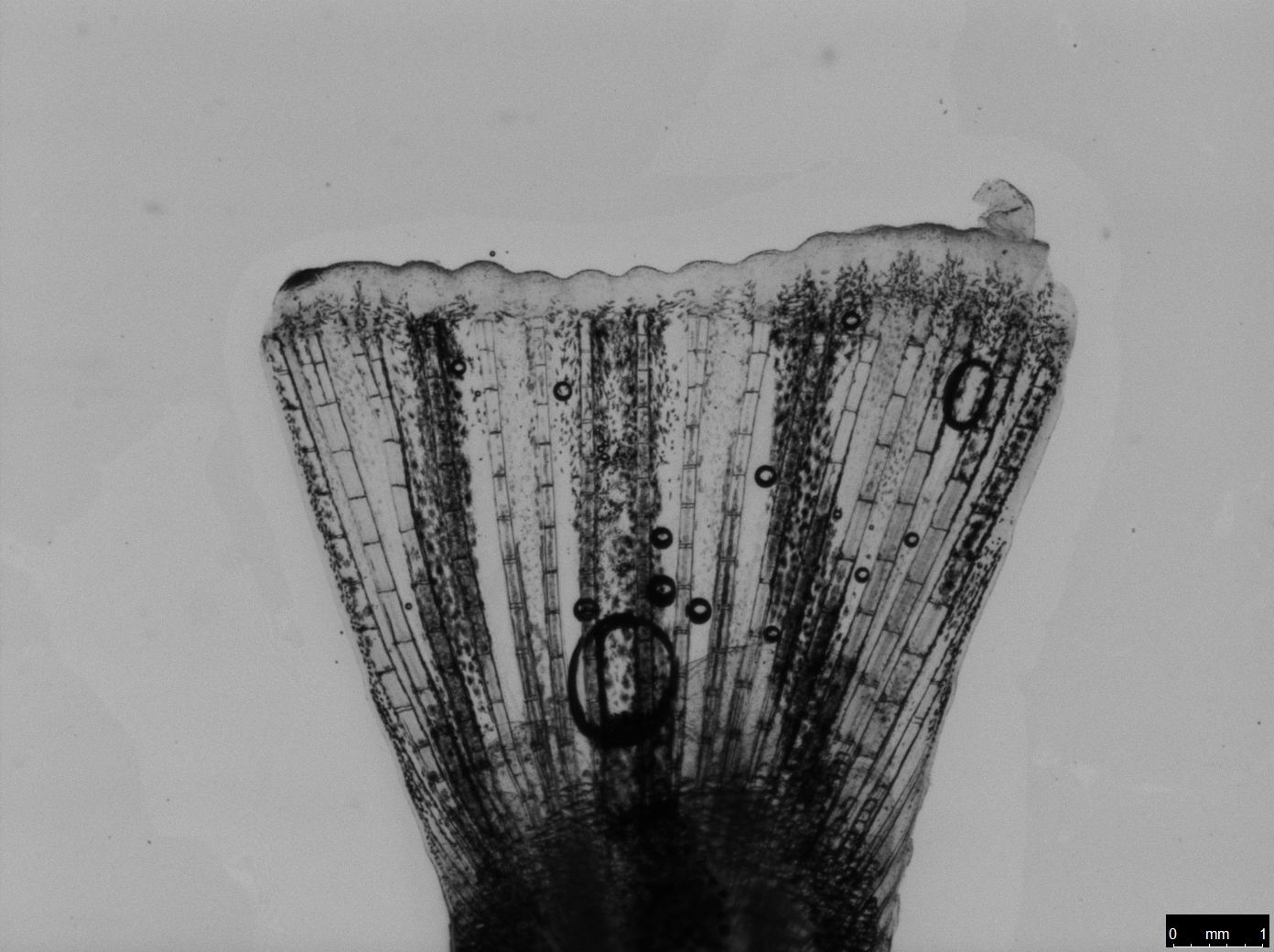

Supplement: Supplementary file 6 — Source data Fig. 5 [file 44319_2025_602_MOESM6_ESM.zip › Figure5/panel D/Mo std 48hpi.jpg]

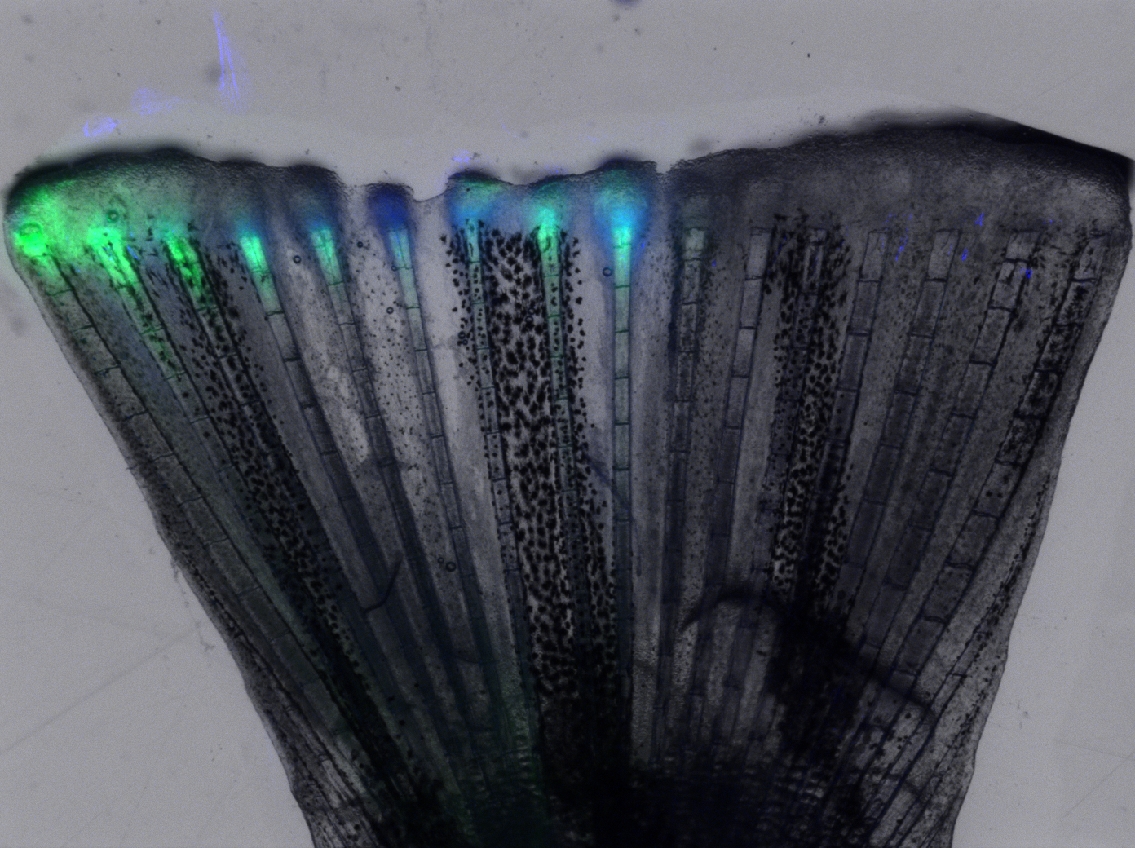

Supplement: Supplementary file 6 — Source data Fig. 5 [file 44319_2025_602_MOESM6_ESM.zip › Figure5/panel D/tert art0dpi.jpg]

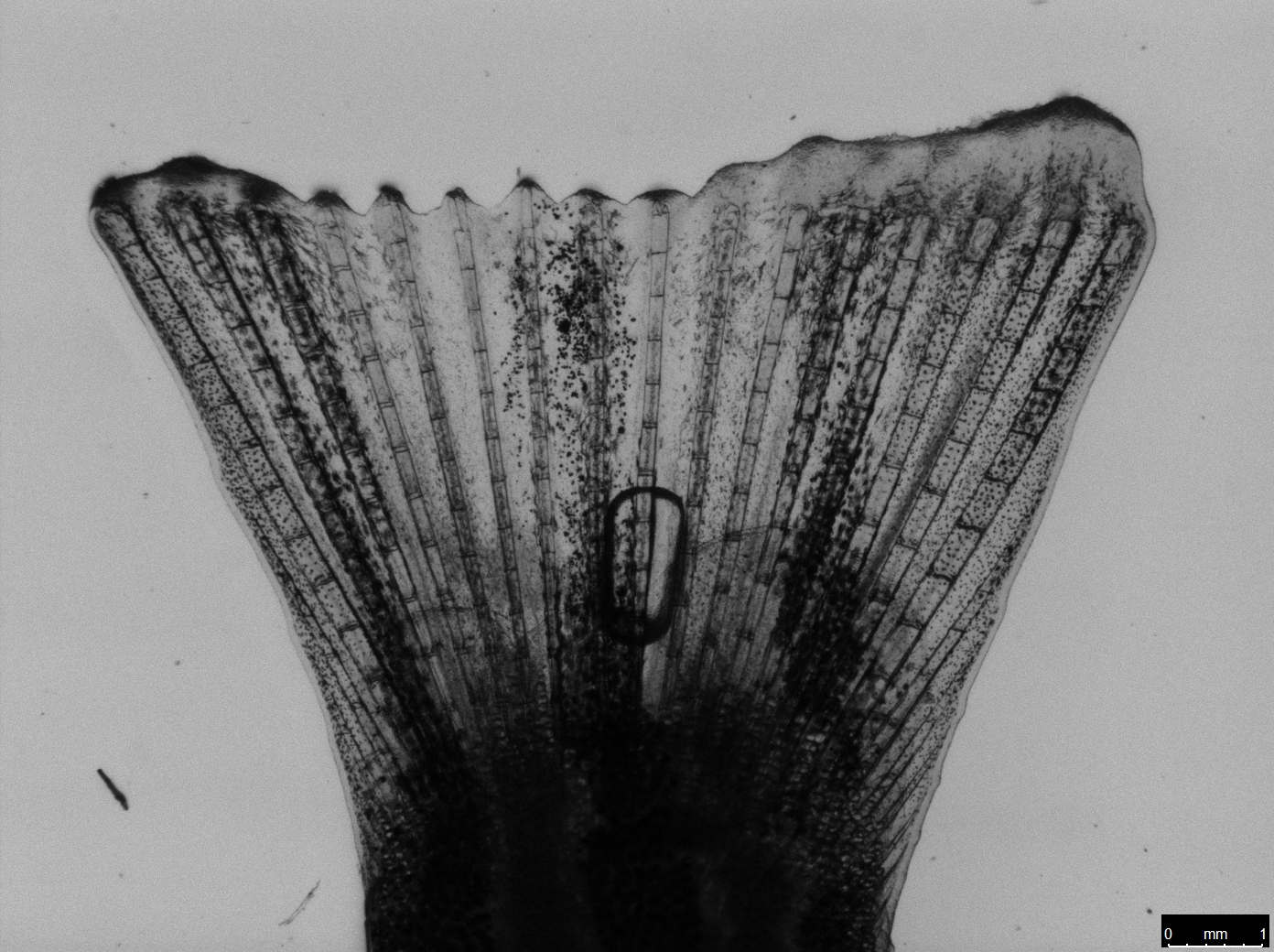

Supplement: Supplementary file 6 — Source data Fig. 5 [file 44319_2025_602_MOESM6_ESM.zip › Figure5/panel D/Mo atr tert 48hpi.jpg]

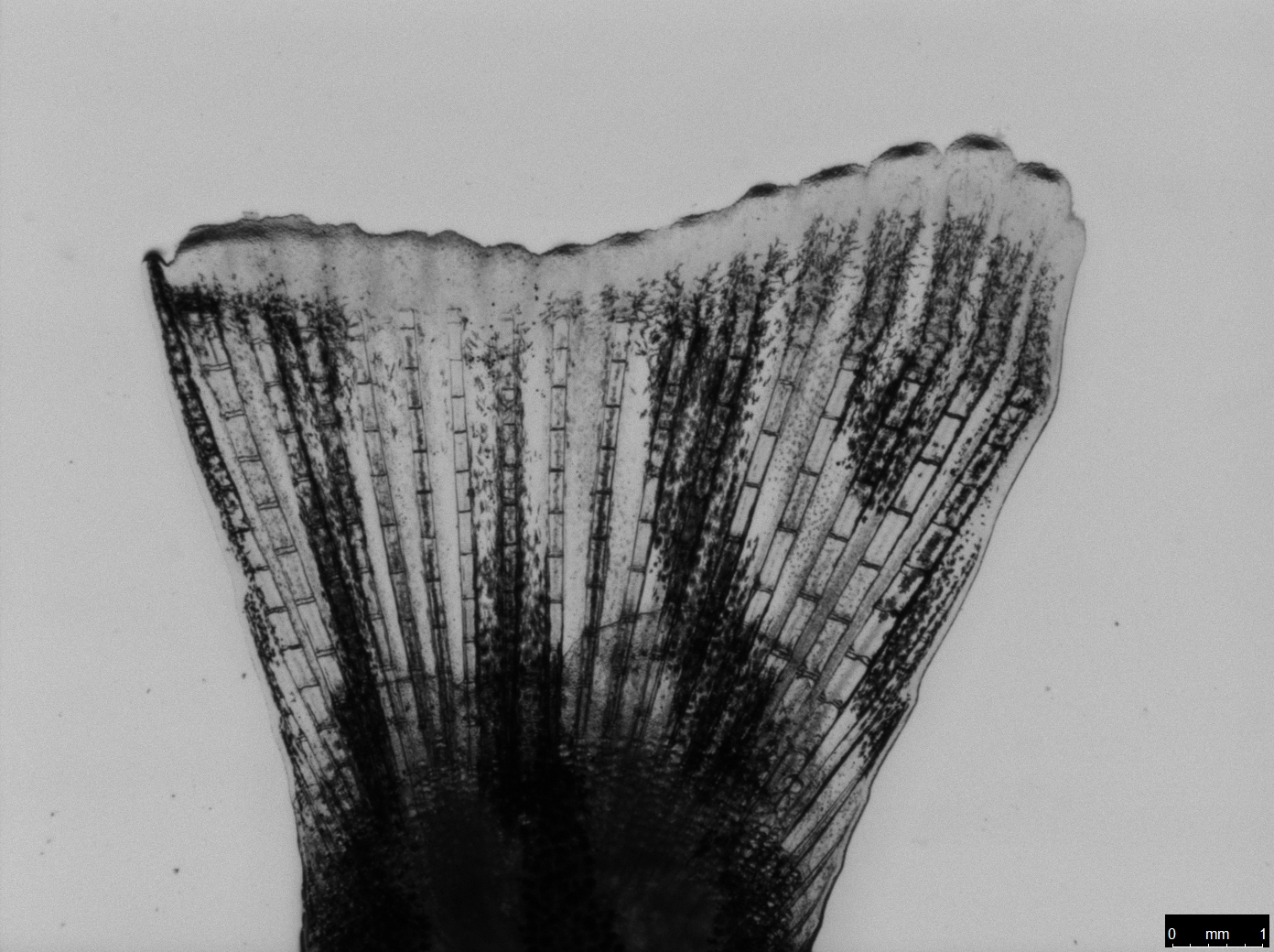

Supplement: Supplementary file 6 — Source data Fig. 5 [file 44319_2025_602_MOESM6_ESM.zip › Figure5/panel D/Mo atr 48hpi.jpg]

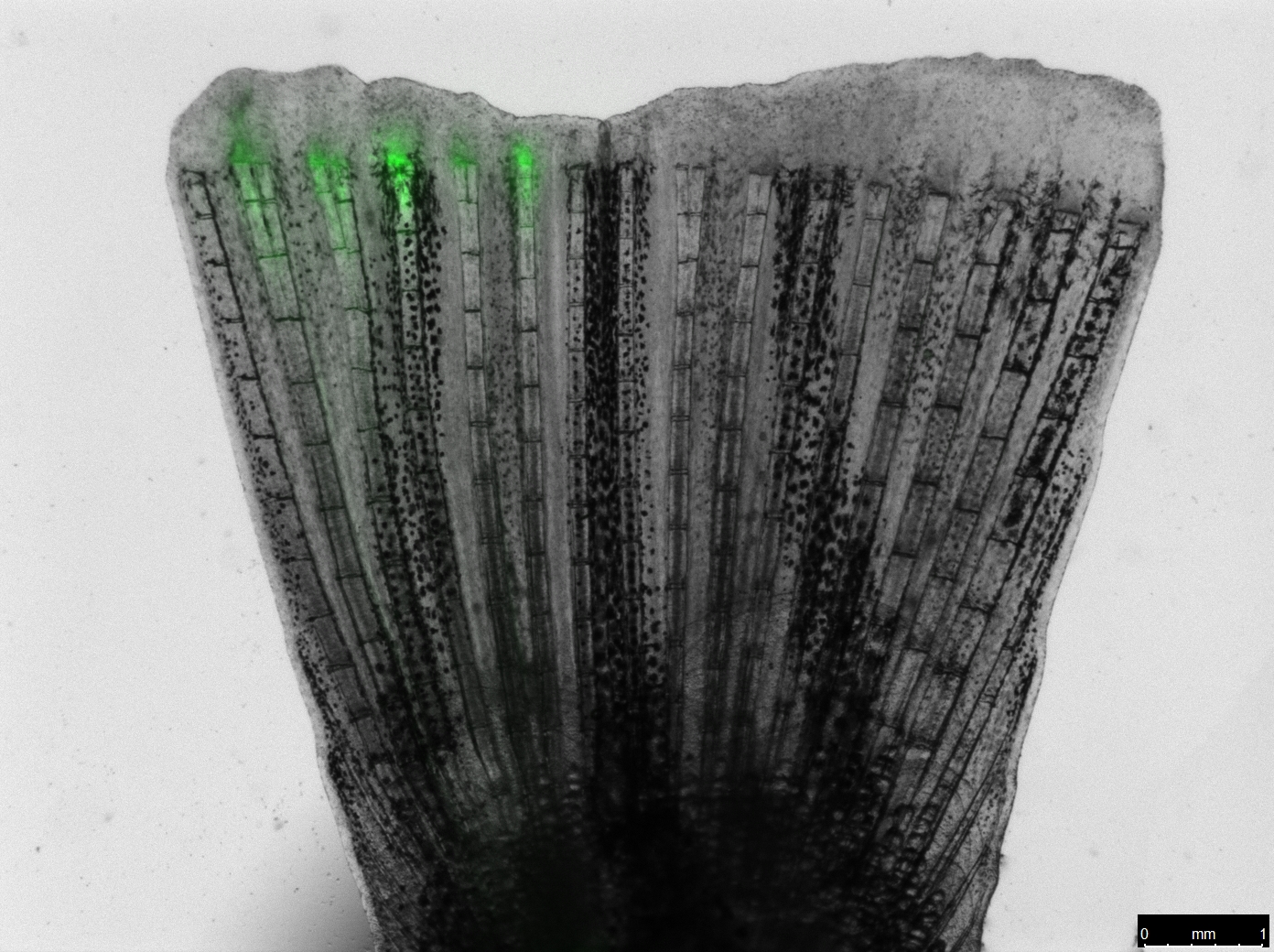

Supplement: Supplementary file 6 — Source data Fig. 5 [file 44319_2025_602_MOESM6_ESM.zip › Figure5/panel D/Mo std 0hpi.jpg]

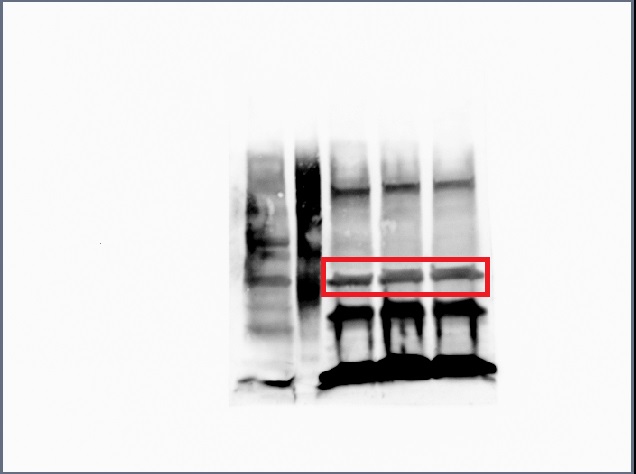

Supplement: Supplementary file 7 — Source data Fig. 6 [file 44319_2025_602_MOESM7_ESM.zip › Figure6/panel B/actin.jpg]

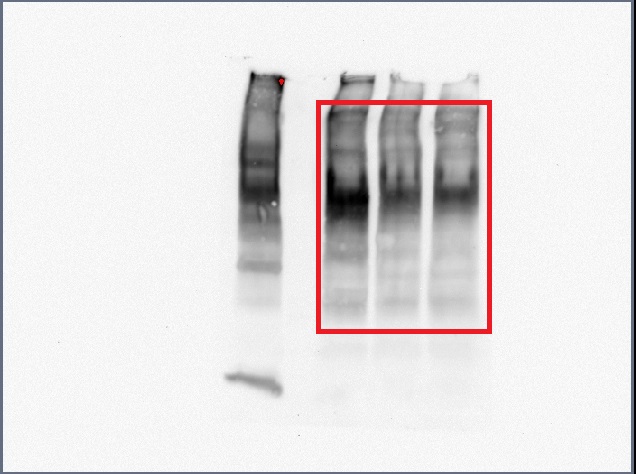

Supplement: Supplementary file 7 — Source data Fig. 6 [file 44319_2025_602_MOESM7_ESM.zip › Figure6/panel B/p-ATR-ATM substrate.jpg]

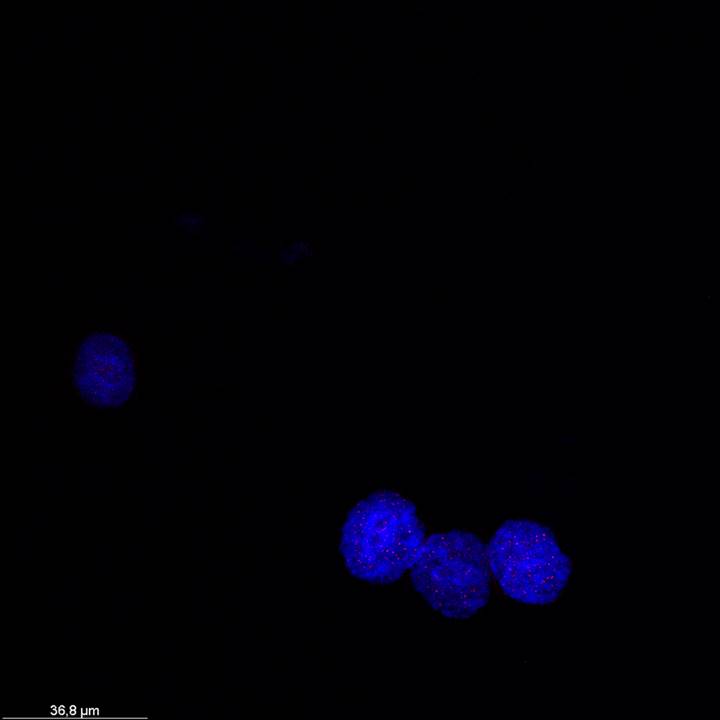

Supplement: Supplementary file 8 — All Figures EV Source Data [file 44319_2025_602_MOESM8_ESM.zip › FigureEV1/panel B/left.jpg]

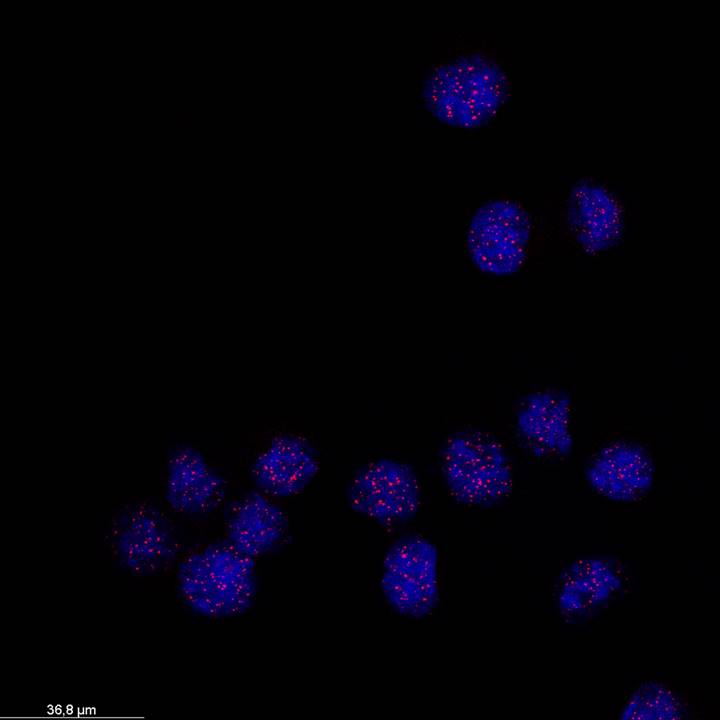

Supplement: Supplementary file 8 — All Figures EV Source Data [file 44319_2025_602_MOESM8_ESM.zip › FigureEV1/panel B/right.jpg]

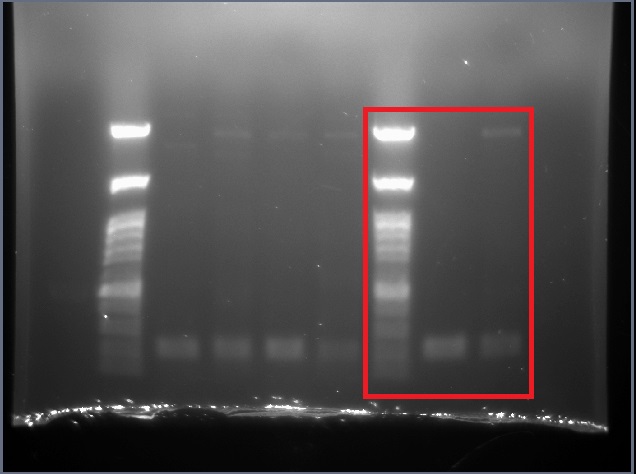

Supplement: Supplementary file 8 — All Figures EV Source Data [file 44319_2025_602_MOESM8_ESM.zip › FigureEV2/panel B/Gel.jpg]

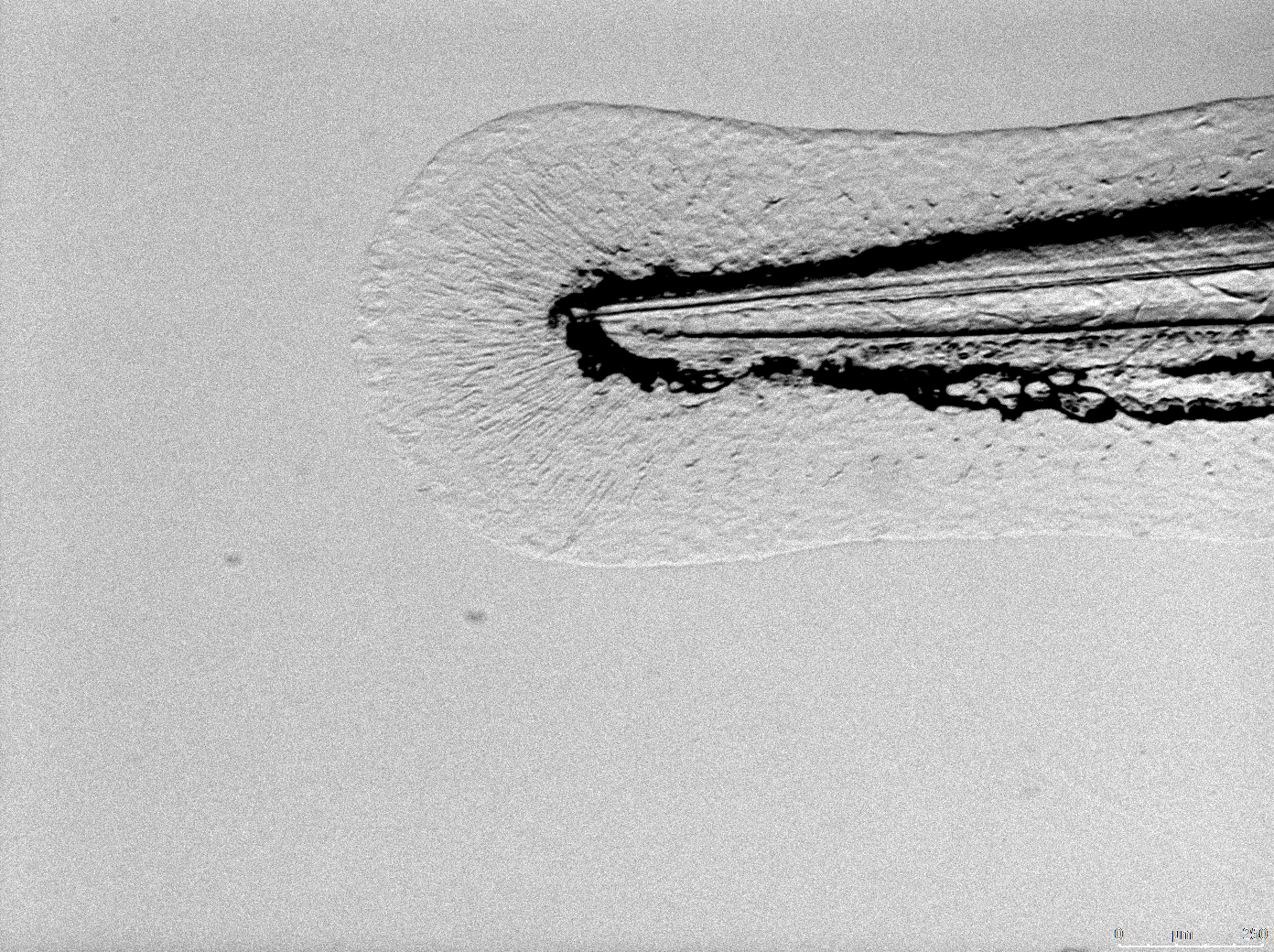

Supplement: Supplementary file 8 — All Figures EV Source Data [file 44319_2025_602_MOESM8_ESM.zip › FigureEV5/panel B/up left.tif]

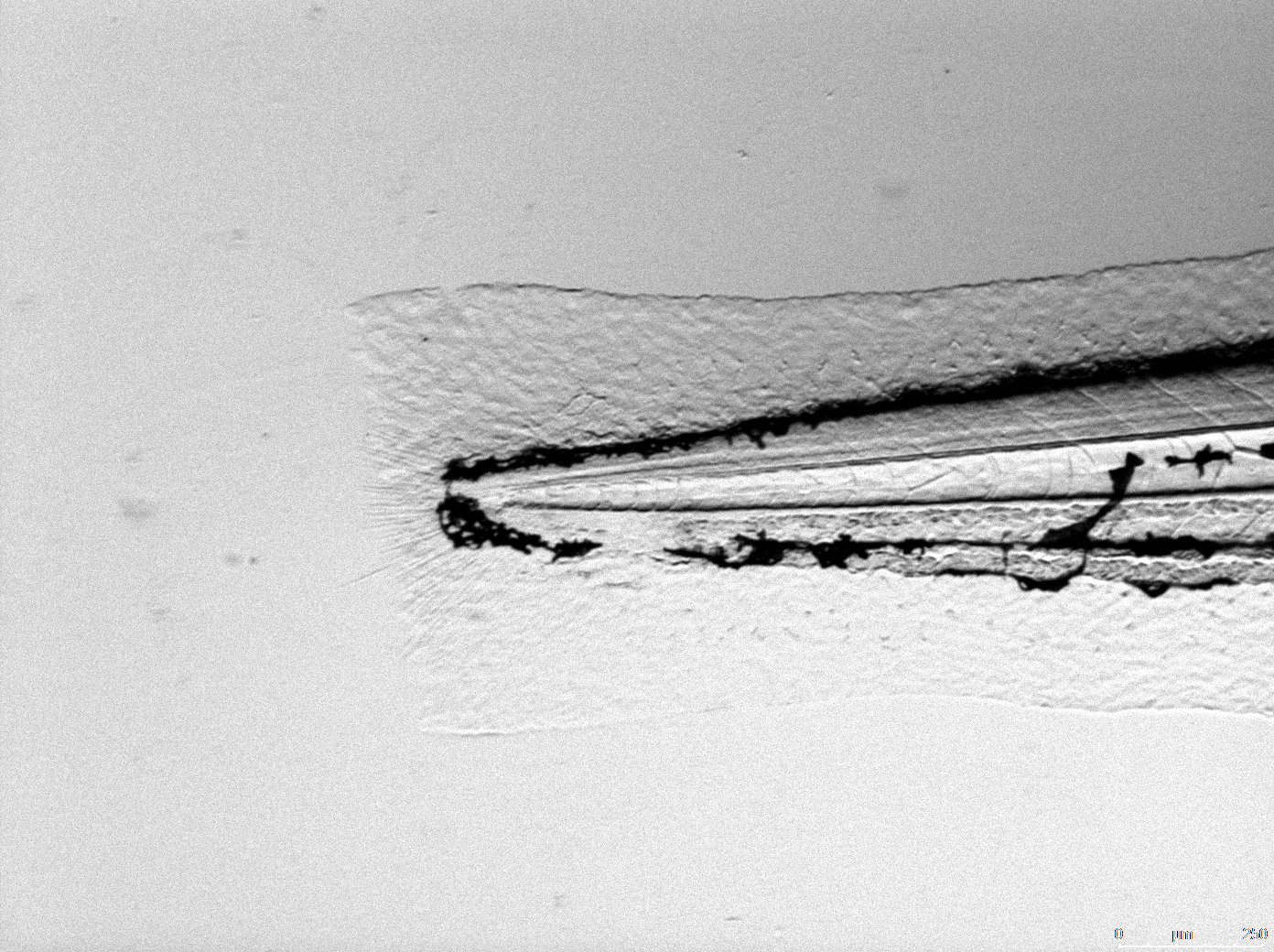

Supplement: Supplementary file 8 — All Figures EV Source Data [file 44319_2025_602_MOESM8_ESM.zip › FigureEV5/panel B/down middle.tif]

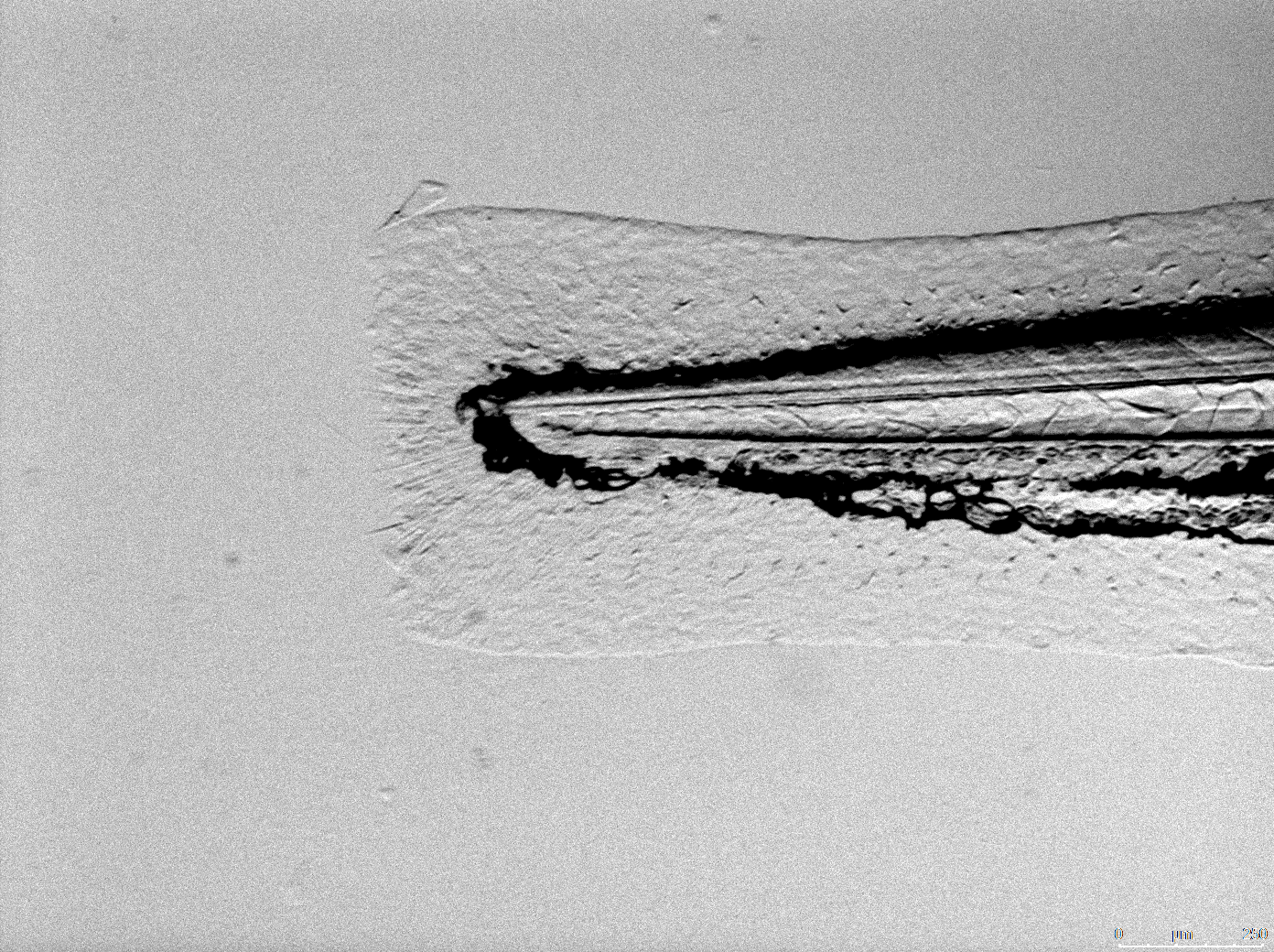

Supplement: Supplementary file 8 — All Figures EV Source Data [file 44319_2025_602_MOESM8_ESM.zip › FigureEV5/panel B/up middle.tif]

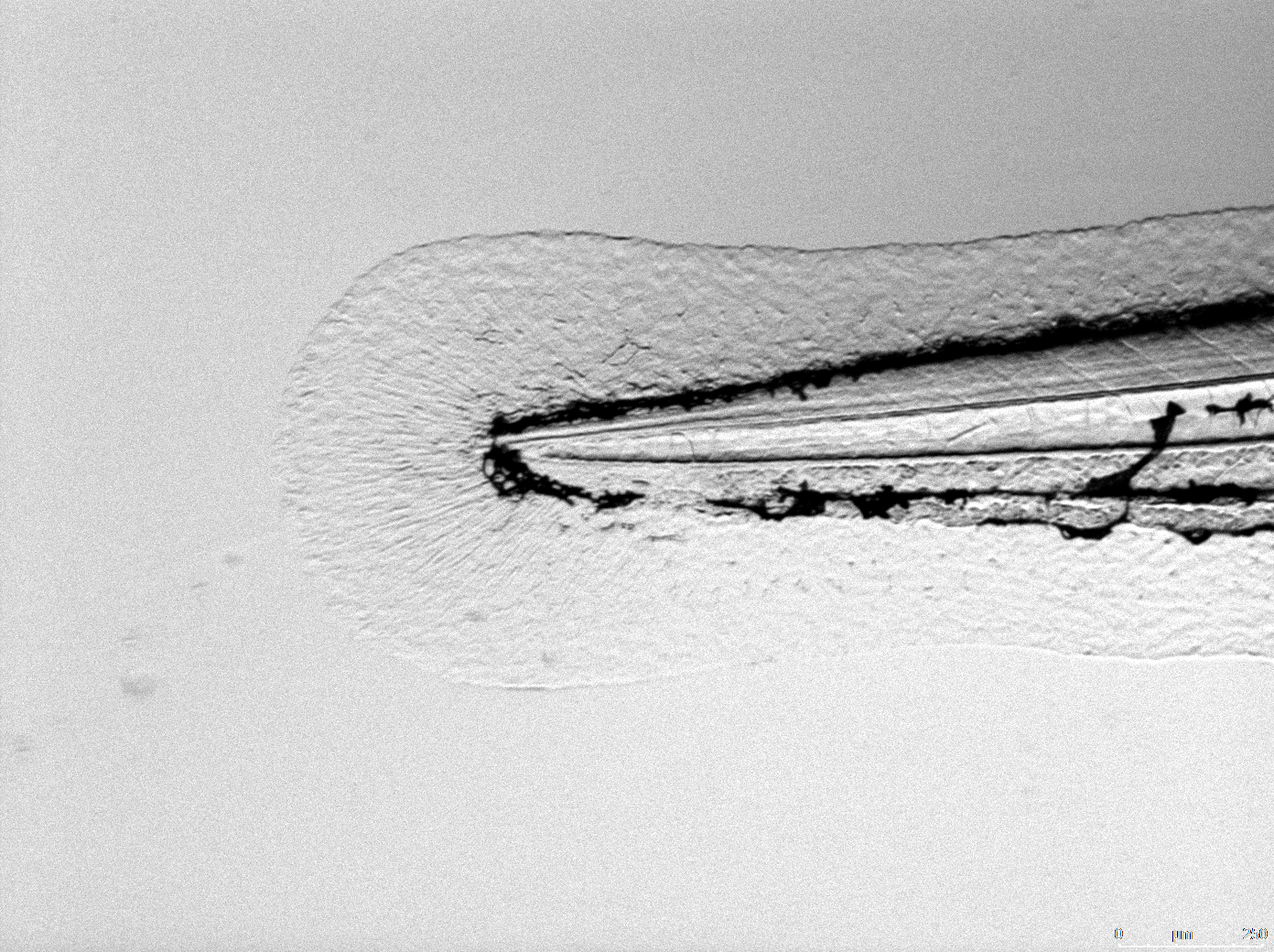

Supplement: Supplementary file 8 — All Figures EV Source Data [file 44319_2025_602_MOESM8_ESM.zip › FigureEV5/panel B/down left.tif]

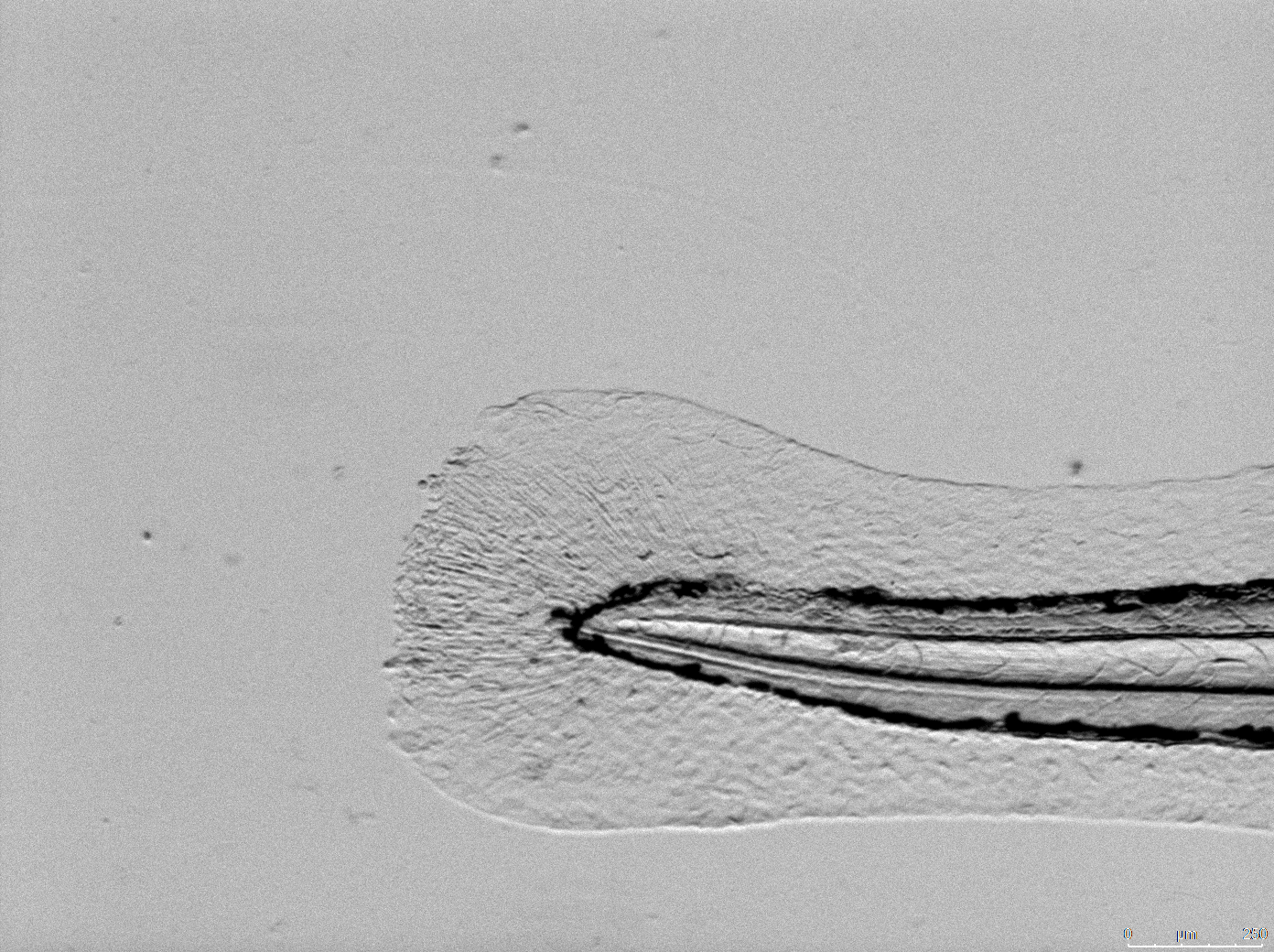

Supplement: Supplementary file 8 — All Figures EV Source Data [file 44319_2025_602_MOESM8_ESM.zip › FigureEV5/panel B/up right.tif]

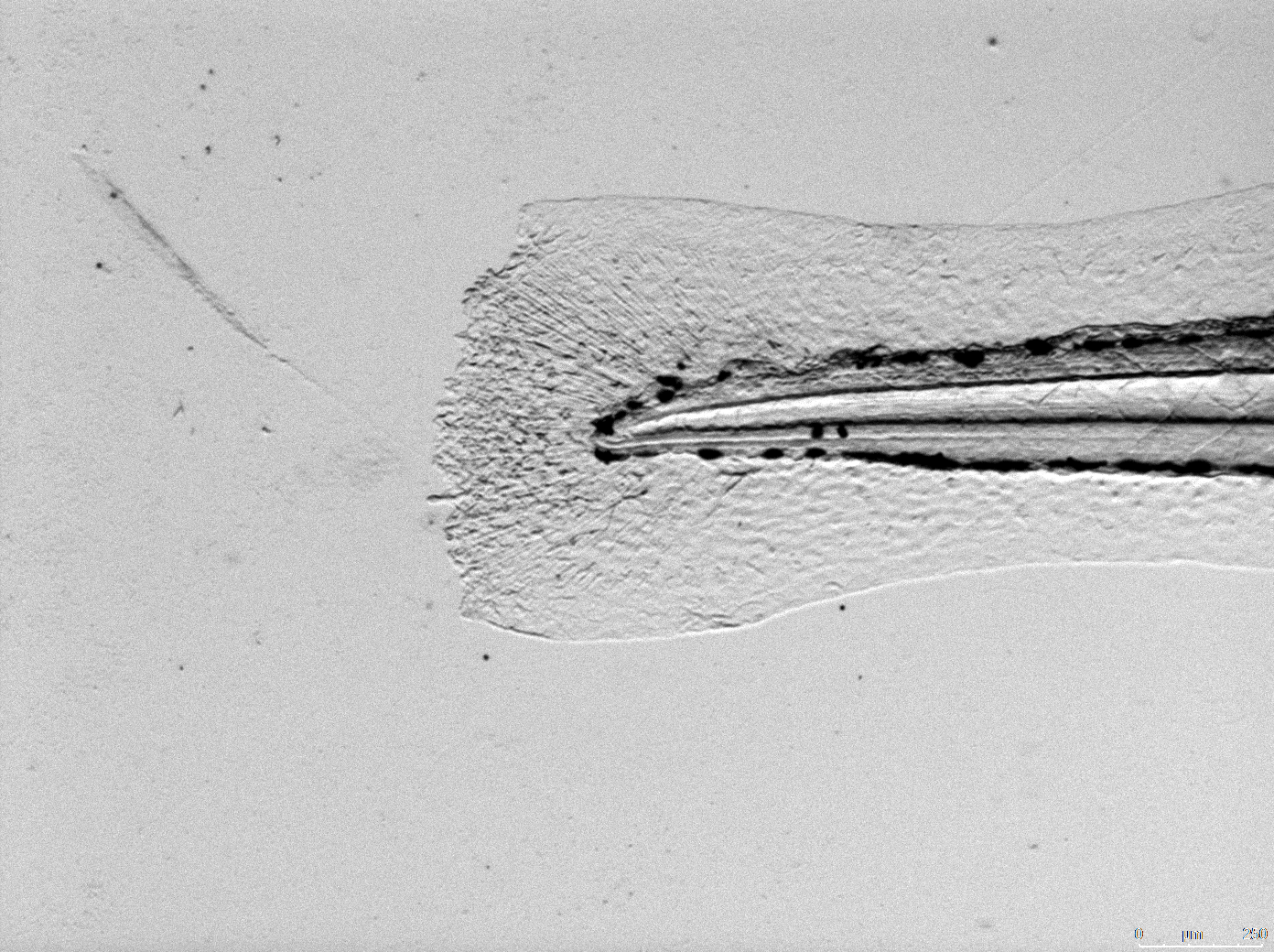

Supplement: Supplementary file 8 — All Figures EV Source Data [file 44319_2025_602_MOESM8_ESM.zip › FigureEV5/panel B/down right.tif]
