# Supplementary material for: Telomerase and alternative lengthening of telomeres coexist in the regenerating zebrafish caudal fins
Source: EMBO Rep. 2025 Oct 21;26(23):5776–98. doi: 10.1038/s44319-025-00602-6 (PMC12678820; doi:10.1038/s44319-025-00602-6)
Supplement: Supplementary file 9 — Expanded View Figures [file 44319_2025_602_MOESM9_ESM.pdf]

Expanded View Figures

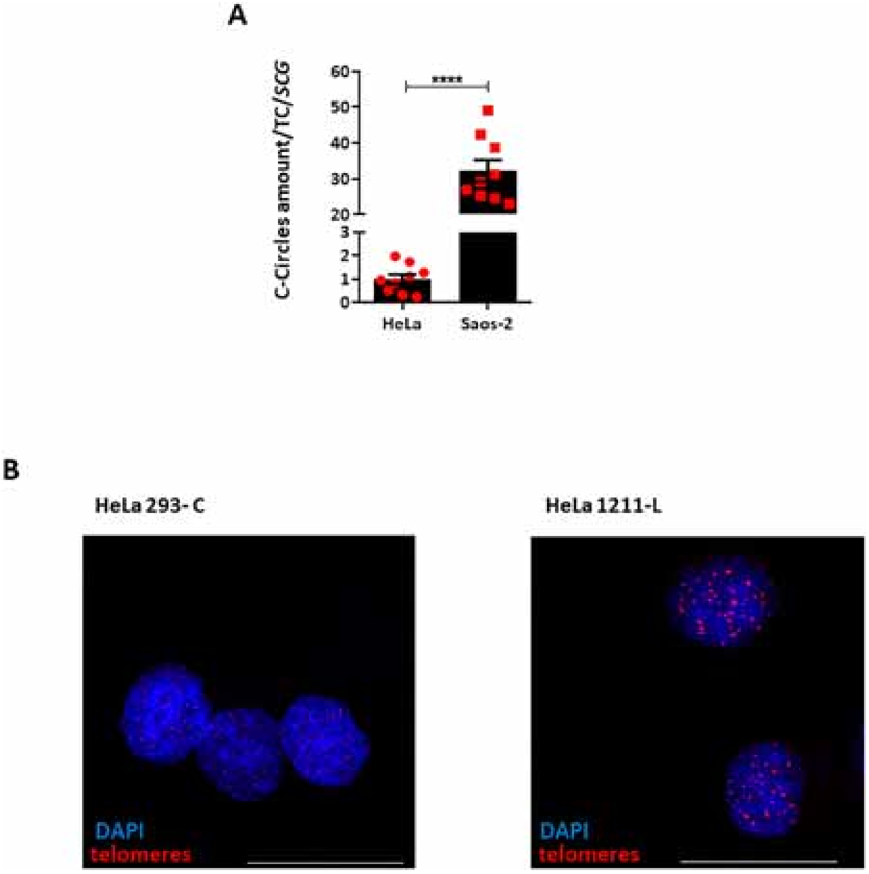

Figure EV1. C-circles abundance and telomere Q-FISH in control cell lines.

(**A**) C-circles abundance in ALT-negative human cervical tumor cell line (HeLa) and ALT-positive human osteosarcoma tumor cell line (Saos-2). $n = 3$ experiments with three technical replicates each. (**B**) Q-FISH in HeLa cells. HeLa 293 has a telomere length of 2.7 kb, and HeLa 1211 has a telomere length of 23 kb. Blue=DAPI, red=telomere. Scale bar, 36.8 μm. Data were mean + s.e.m. ****$p < 0{,}0001$ for the Mann–Whitney test. TC telomere content, SCG single-copy gene. When exact p values are not indicated is because GraphPad Prism software does not show it. Source data are available online for this figure.

 © The Author(s)

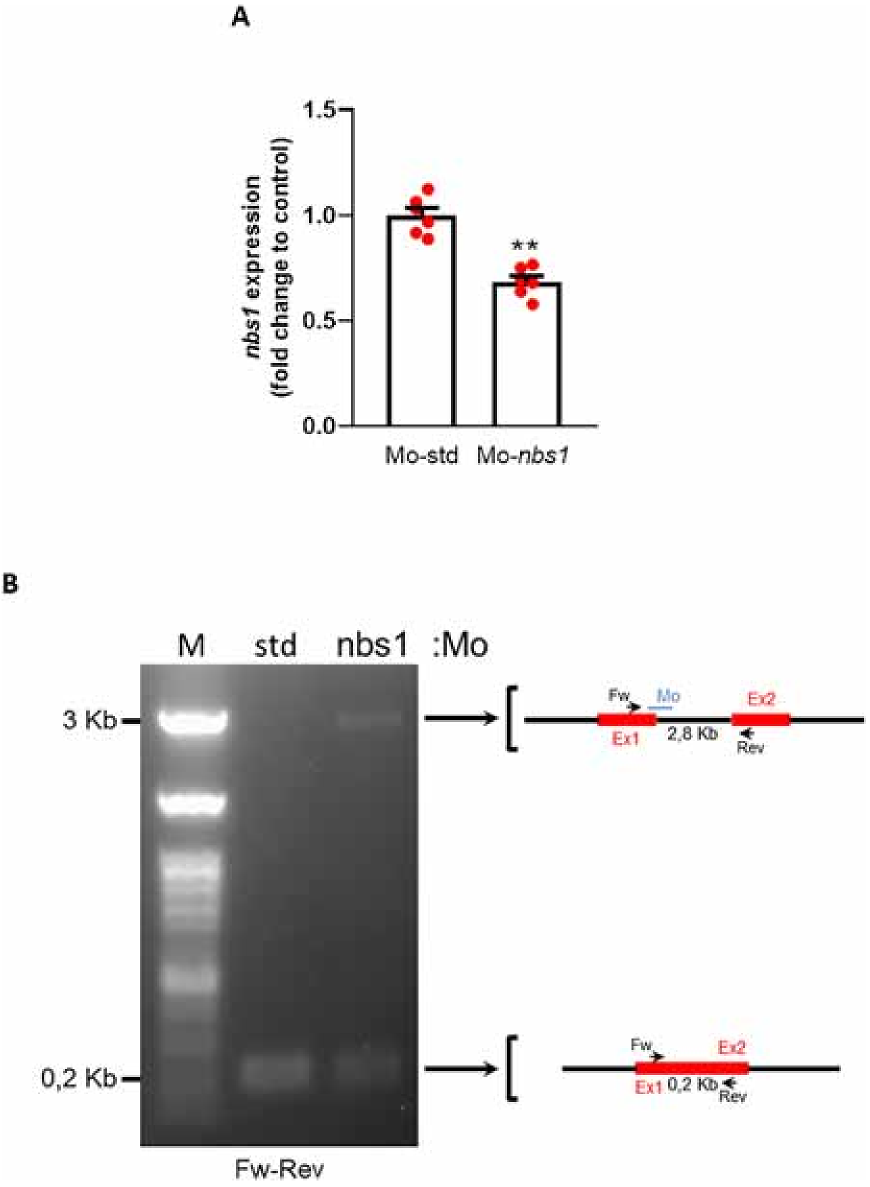

Figure EV2. Efficiency of the nbs1 morpholino.

(**A**) qPCR showing the expression of nbs1 in 3dpf larvae upon microinjection of standard (Mo-std) or nbs1 (Mo-nbs1) morpholinos. $n = 3$ experiments with two technical replicates each. (**B**) schematic diagram and PCR showing the retention of the intron 1 of the zebrafish nbs1 gene when the morpholino is used, and the reduction in the amount of the mRNA species splicing the intron. Data were mean ± s.e.m. $p = 0{,}0022$ for Mann–Whitney test. When exact p values are not indicated is because GraphPad Prism software does not show it. Source data are available online for this figure.

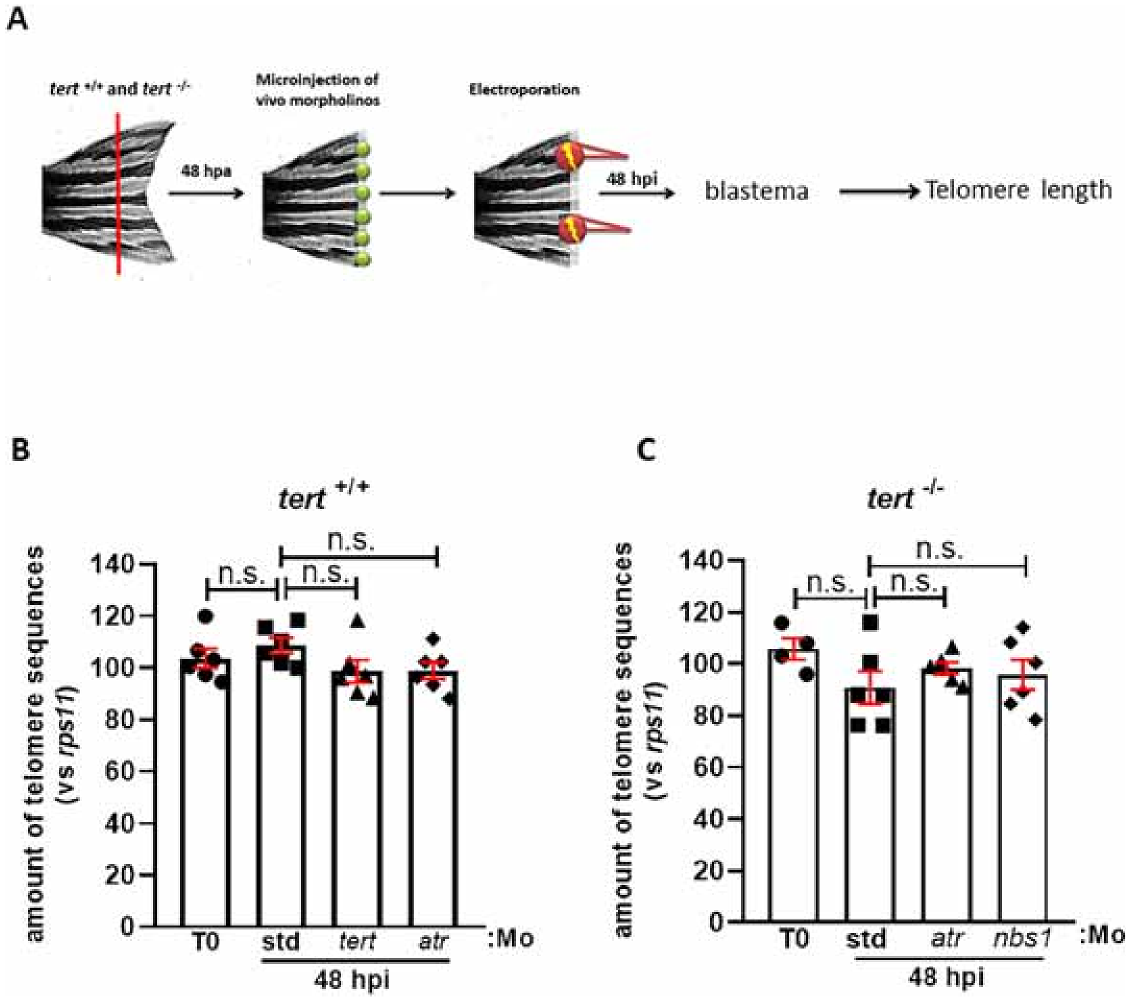

Figure EV3. Telomere length is maintained in regenerating tissue of wild-type and tert mutants upon morpholino injection.

(**A**) Schematic workflow of the experiment. (**B**) Quantification by qPCR of telomere length per single-copy gene (rps11) in the regenerated tissue of tert+/+ and (**C**), tert −/− fish after 48 h post the injection of the indicated morpholinos. T0 is the fin tissue amputated. $n = 3$ experiments. Each dot represents a biological replicate. Data were mean ± s.e.m. n.s. not significant in one-way ANOVA and Bonferroni´s post hoc test. Source data are available online for this figure.

 © The Author(s)

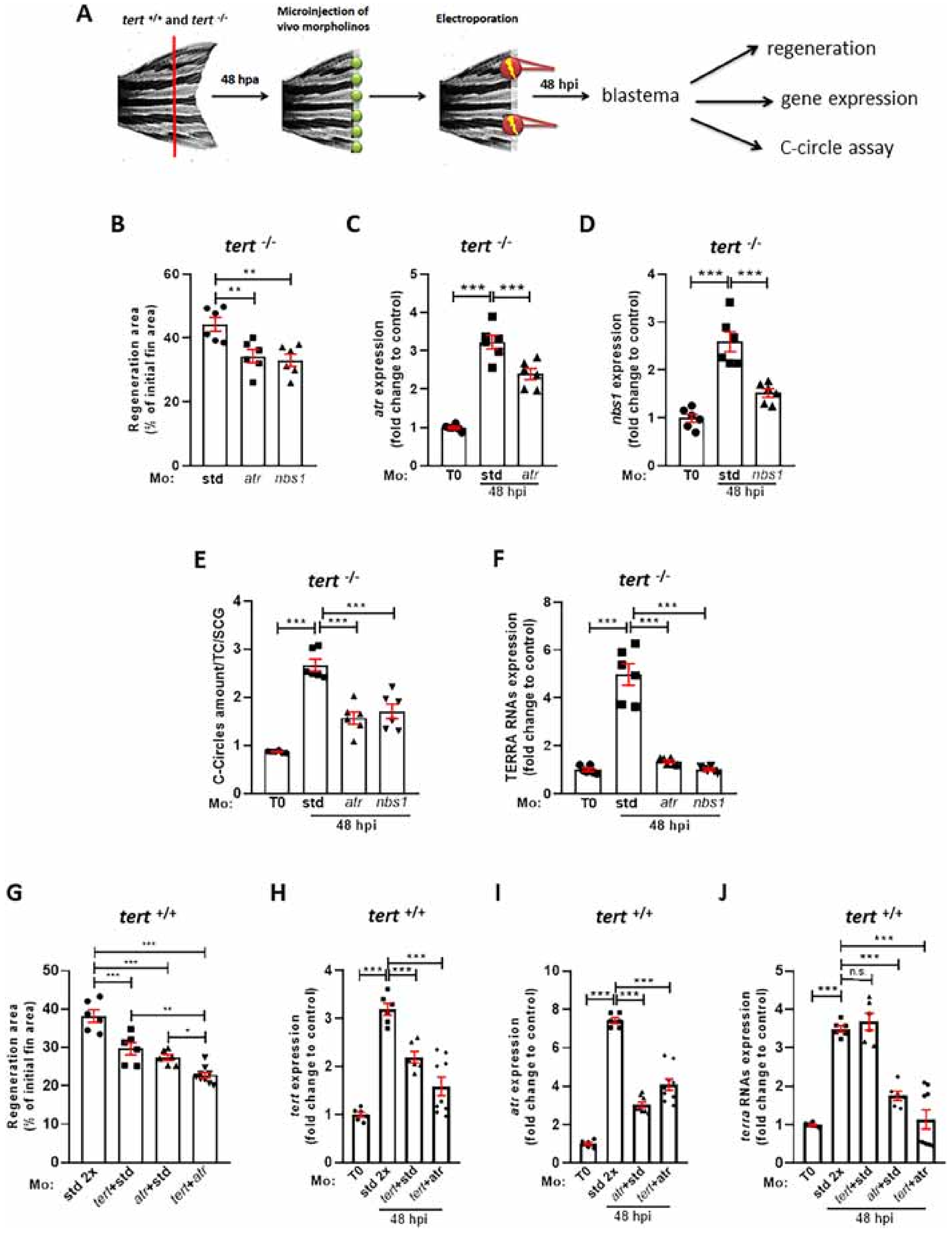
A
tert +/+ and tert -/-
Microinjection of vivo morpholinos
Electroporation
48 hpa
48 hpi
blastema
regeneration
gene expression
C-circle assay
B
tert -/-
Regeneration area (% of initial fin area)
Mo: std atr nbs1
C
tert -/-
atr expression (fold change to control)
Mo: T0 std atr
48 hpi
D
tert -/-
nbs1 expression (fold change to control)
Mo: T0 std nbs1
48 hpi
E
tert -/-
C-Circles amount/TC/SCG
Mo: T0 std atr nbs1
48 hpi
F
tert -/-
TERRA RNAs expression (fold change to control)
Mo: T0 std atr nbs1
48 hpi
G
tert +/+
Regeneration area (% of initial fin area)
Mo: std 2x tert+std atr+std tert+atr
H
tert +/+
tert expression (fold change to control)
Mo: T0 std 2x tert+std tert+atr
48 hpi
I
tert +/+
atr expression (fold change to control)
Mo: T0 std 2x atr+std tert+atr
48 hpi
J
tert +/+
terra RNAs expression (fold change to control)
n.s.
Mo: T0 std 2x tert+std atr+std tert+atr
48 hpi

Figure EV4. Regeneration capacity and ALT mechanism depends on atr and nbs1 in zebrafish caudal fins.

(**A**) Schematic diagram of the workflow of the experiment. (**B**) Regeneration area of caudal fin in tert deficient animals 48 hpa microinjected with the indicated morpholinos. $n = 3$ experiments. Each dot represents a biological replicate. Exact p values are $p = 0.0067$ std vs atr and $p = 0.0027$ std vs nbs. (**C**, **D**) qPCRs showing an efficient gene expression downregulation by morpholino injection. $n = 3$ experiments. Each dot represents a biological replicate. Exact p value in (**C**) is $p = 0.001$ for std vs atr. (**E**, **F**) C-circles amount per telomere content (TC) and single-copy gene (SCG) and TERRA RNA levels in regenerated blastema of tert$-/-$ fish microinjected with the indicated morpholinos. $n = 3$ experiments. Each dot represents a biological replicate. (**G**) Regeneration area of caudal fin in wild-type animals 48 hpa microinjected with double dose of standard (std 2x), tert + std, atr + std, or tert + atr morpholinos. $n = 3$ experiments. Each dot represents a biological replicate. (**H**–**J**), qPCRs showing an efficient gene expression downregulation by morpholino injection, and TERRA RNA levels. $n = 3$ experiments. Each dot represents a biological replicate. T0 is the fin tissue amputated. Data were mean ± s.e.m. $^{**}P < 0.01$ and $^{***}P < 0.001$ for one-way ANOVA plus Bonferroni's post hoc test. n.s. not significant. When exact p values are not indicated is because GraphPad Prism software does not show it. Source data are available online for this figure.

 © The Author(s)

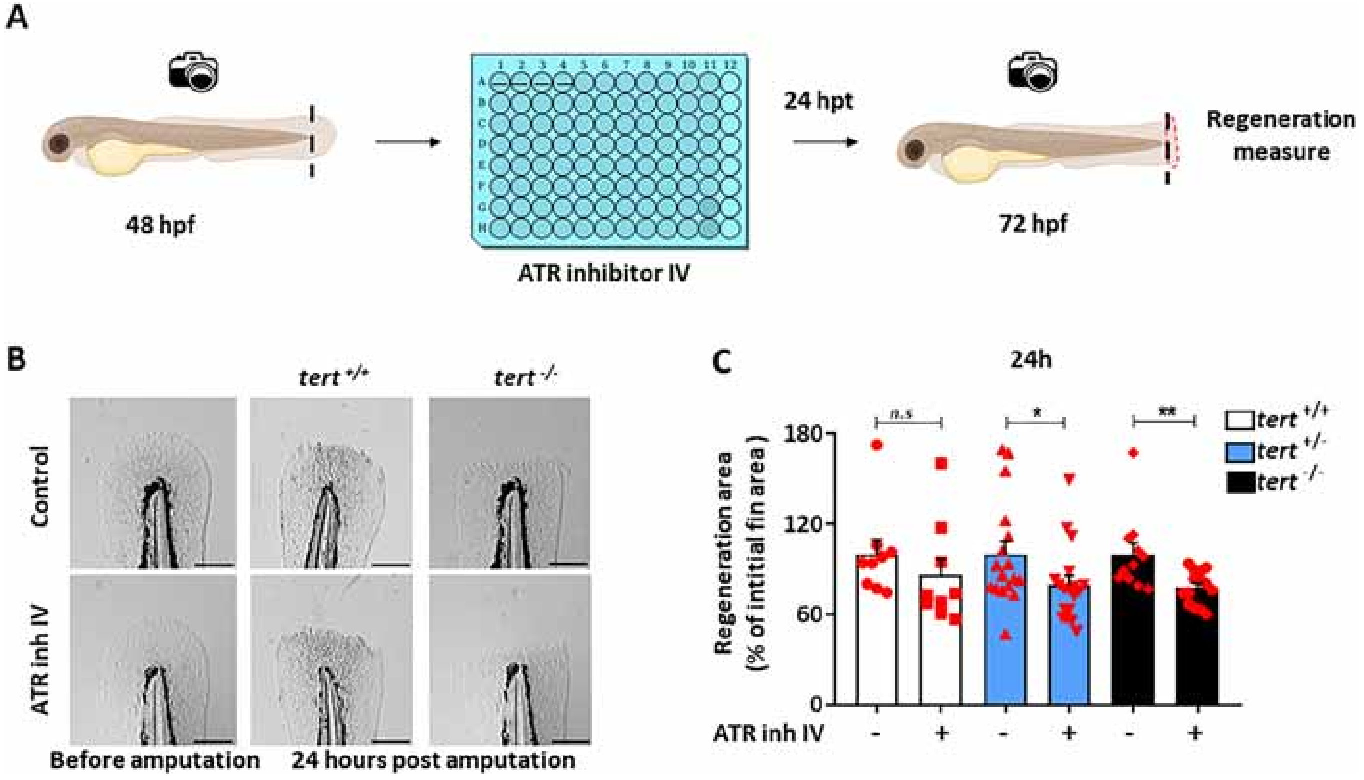

Figure EV5. Determination of the effect of several concentrations of ATR Inhibitor IV on zebrafish larvae.

(**A**) Schematic diagram of the workflow of the experiment. (**B**) Representative pictures of regenerated caudal fin of the larvae 24 h post amputation treated or not with ATR inhibitor IV. Scale bar, 250 μm. (**C**) Regeneration of caudal fin of the larvae 24 h post amputation treated or not with ATR inhibitor IV in the three different *tert* genotypes. $n = 3$ experiments. Each dot represents a biological replicate. Data in (**C**) is mean + s.e.m. Exact p values are $p = 0.042$ in $tert^{+/-}$ and $p = 0.0049$ in $tert^{-/-}$ for unpaired t-test between treated and untreated. Source data are available online for this figure.
